# A novel treatment and derivatization for quantification of residual aromatic diisocyanates in polyamide resins

**DOI:** 10.1038/s41598-022-17316-7

**Published:** 2022-07-29

**Authors:** Genny Pastore, Serena Gabrielli, Ezio Leone, Manuela Cortese, Dario Gentili, Giovanna Biondi, Enrico Marcantoni

**Affiliations:** 1 School of Science and Technology, University of Camerino, ChIP Building, Via Madonna delle Carceri, 62032 Camerino, Italy; 2 Elantas Europe S.R.L., Altana Group DE, Zona Ind.le Campolungo 35, 63100 Ascoli Piceno, Italy

**Keywords:** Polymer chemistry, Polymer characterization

## Abstract

In the scientific context, the environmental and healthy impact of polymers is more related to the residual monomer content rather than their macromolecular structure, due to the monomer capability to interact with membrane cells. For this a novel method to stabilize and quantify residual monomeric isocyanates in high thermal resistance polyamide resins (PAs) has been developed. This new analytical method resulted in an improvement concerning the quantification of residual aromatic diisocyanates in viscous polymeric matrices by using a simple and cheap technique like HPLC-VWD. Diisocyanate monomers were derivatized with dibutylamine, resulting in stable urea derivatives that were simultaneously analysed and quantified. The method was applied to solvent-based polyamide resins, used as primary electrical insulation, for avoiding additional step of solvent removing before the analysis. The quantification of residual monomers answers to the provisions imposed by European Regulation N. 1907/2006 (REACH) for polymer registration, and the necessity of an early evaluation of the occupational risk associated with the use of diisocyanates, due to their toxicity and high reactivity towards moisture.

## Introduction

**Figure 1:**
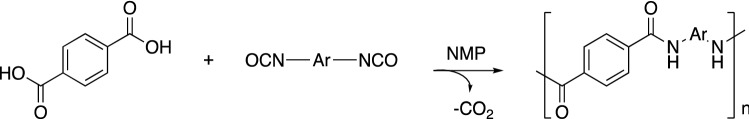
General scheme for polyamide synthesis.

Polyamides (PAs) are versatile products for a broad range of commercial applications due to their unique thermal, mechanical, and electrical properties^1^. Such polymeric materials are among the most important and useful thermoplastics made from monomers capable of giving linkages in the main polymer chain that provide mechanical strength and barrier properties^2^. They are important in engineering because they offer high performance at a reasonable cost, and then they are considered as one of the most versatile engineering plastics. The polyamide resins under investigation are synthesized from conventional condensation polymerizations, which proceed through a step-growth mechanism of para-substituted AB-type aromatic monomers ^3^. They show the ability to control molecular weights and dispersities of condensation polyamides, and in particular they are prepared from a variety of aromatic diisocyanates (DIs) and therephthalic acid in a polar aprotic solvent (N-methylpyrrolidone, NMP) as shown in Fig. 1^4–6^. The attractive features of this reaction include (1) the use of reactants which are tolerant of small deviations in stoichiometric equivalence, (2) easy remove of the volatile condensate such as carbon dioxide, (3) high yields of polymer, and (4) generation of a wide range of aromatic polyamides.

**Figure 2:**
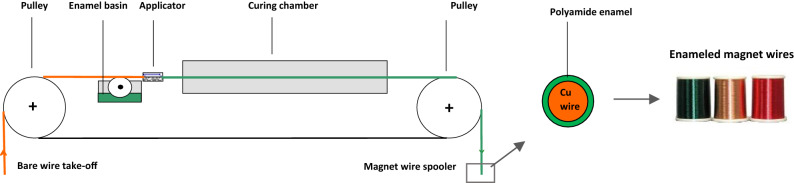
Schematic representation of the general coating process.

The most common diisocyanates used are the mixture of isomers 2,4-, 2,6-toluene diisocyanates (TDI) and 4,4’-methylene diphenyl diisocyanate (MDI), which are both aromatic. The final product is a solvent-based varnish with a viscosity range between 1000 and 3000 mPa∙s, which is applied and cured at high temperature on copper wires. (Fig. 2). MDI and TDI have different chemical behaviour and confer different mechanical properties to the final product. They can be used separately, but also as a combination of the two.

The industrial polymerization process reveals a lack of reproducibility and the need to add an additional amount of aromatic diisocyanates on a case-by-case basis.

At the same time, European Regulation N. 1907/2006 (REACH) imposes to the companies the registration of residual monomers, if they are present in concentration over 2% w/w, and if the monomers amount is totally over 1 ton per year. This legal obligation goes together with the ethical obligation to evaluate the residual TDI or MDI, due to their toxicological issues: isocyanates exposure is associated with occupational hazards^7–9^. In fact, in the case of wire enamel polyamides, wire enamelling workers are those who are potentially at risk to diisocyanates exposure during the curing process which occurs at high temperatures during the normal production activities^10^. In addition, residual aromatic diisocyanates react with moisture, which is always present in hygroscopic solvents, giving rise to many products, including urea, oligoureas, polyureas, carbon dioxide, and aromatic amines^11,12^, which are known to be mutagenic and are listed as suspected or possible human carcinogens^13–15^. These residual aromatic amines, 4,4′-methylenedianiline, could decompose with formation of aniline when heated at high temperatures in enamelling ovens during the curing process^2^.

Work-related exposure to DIs can occur during the production and raw material treating, as well as in further handling with the finished products, especially in the activities concerning the general coating process which is performed at high temperature. Understanding the degradation mechanism of polymeric materials that could lead to the formation of dangerous products for the environment is particularly useful in combating the global problem of pollution due to polymer-matrix composite materials^16^. The minimization of residual monomer content is a priority as residual monomers can result in increased hazards while representing decreased production efficiency and increased costs. It should be noted that when these low molecular weight species are considered, residual monomer must be included, but excluding other components such as additives or impurities^17^.

Most of the scientific literature concerning diisocyanates analysis, covers the determination of them in air in industrial environments^18–20^. Numerous methods were published that detail isocyanates analysis in polymer matrices by using fast infrared spectroscopic techniques, such as MIR and NIR^21^, but these methodologies are widely used to monitor high concentration of diisocyanates, so they are not suitable for PAs due to the low concentration of residuals. Another interesting analytical technique is the nuclear magnetic resonance spectroscopy of fluorine-19 (^19^F-NMR)^22^, usable modifying the isocyanate derivative with 1,1,1,3,3,3-hexafluoro isopropyl alcohol (HFIP). Unfortunately, also in this case, quantitative magnetic resonance technique (qNMR) proves to be inaccurate due to the matrix complexity. Suitable techniques for very complex matrices, which allow determining such a low concentration of analytes, are chromatographic ones^23^. In the most suitable scientific paper found for the case under study, the authors used two techniques to determine MDI in a polyurethane foam matrix^24^: reversed-phase high performance-liquid chromatography coupled with ultraviolet detector (HPLC–UV) and reversed-phase high-performance liquid chromatography coupled with mass spectrometry triple quadrupole detector (HPLC–ESI–MS/MS). The first is widespread and easy to use technique, the latter gives a more specific response, but it is more expensive and complex. Since all the aforementioned articles refer to solid or foam polyurethane samples, we were not aware of any existing method for the determination of free monomeric DIs in solvent-based polyamide resins. Moreover, very few and aged papers reported the simultaneous quantification of residual MDI and TDI monomers. Consequently, answering to the legal and ethical obligations, we developed and applied a novel derivatization and extraction method for the monitoring of both TDI and MDI for PA resin samples in the same analysis, at early stage of the entire production process. This new method can detect free monomeric diisocyanates in the order of part per million in a polymer matrix never faced so far by using a simple and cheap technique like HPLC-VWD.

## Materials and methods

### Chemicals and materials

The 4,4′-methylene diphenyl diisocyanate (MDI) and a mixture (about 80:20) of 2,4- and 2,6-toluene diisocyanates (TDI) were provided by Elantas Europe S.r.l. and stored at − 18 °C. Reagent grade triethylamine (TEA), benzylamine (BA) and dibutylamine (DBA) were purchased from Sigma Aldrich. Acetone-d_6_ was purchased from Sigma Aldrich.

HPLC water was purified using the Milli-Q system. HPLC grade acetonitrile was purchased from Sigma Aldrich. Technical grade acetonitrile and n-hexane were purchased from Carlo Erba. Technical grade dry tetrahydrofuran (THF) was purchased from Sigma Aldrich.

Three batches of PA were provided by Elantas Europe S.r.l. Samples have a mean viscosity of 1100 mPa s and a mean dry content of 22.9% (solvent used is NMP). Three samples were chosen corresponding to three batches produced over a year (Table 1). One batch was marked as anomalous as slightly greater quantities of MDI and TDI were added during the condensation step of the synthesis process to achieve final specifications.

**Table 1:** PAs sample provided by Elantas Europe S.r.l.

Sample name	Production date	Abnormality
PA 1	August 2020	Yes
PA 2	January 2021	None
PA 3	April 2021	None

The molecular structures of the standards were confirmed by ^1^H-NMR, recorded on a Varian Mercury plus 400 system at 400 MHz^25,26^.

### Standard preparation

DIs derivatives were synthesized using a Perveen et al. adapted method^27^. Specifically, the monomer (10 mmol) was dissolved in 30 mL of dry THF under nitrogen flow and put in an ice bath. Then, the derivatizing agent (10 mmol) was added dropwise followed by the addition of dry triethylamine (12 mmol). Next, the reaction was stirred for 30 min at 0 °C and additional 30 min at 25 °C. The reaction product precipitated as a white solid due to its insolubility in THF. It was filtered and dried under vacuum. The residual white solid was purified by flash column chromatography (hexane:ethyl acetate 60:40) using Isolera™ (Biotage). Derivatization of analytes were conducted under anhydrous condition due to the strong reactivity of DIs with water. Both MDI and TDI were derivatized with two different amines, benzylamine (BA) and dibutylamine (DBA), to evaluate which resulting urea had an elution time such as not to overlap with the components of the matrix. In particular, MDI/BA-1 and MDI/DBA-3 were obtained from MDI monomer, meanwhile TDI/BA-2 and TDI/DBA-4 were produced starting from TDI monomer (Table 2). The purity of the synthesized standards was evaluated by HPLC-VWD at 254 nm.

**Table 2:** DIs derivatization product obtained with benzylamine and dibutylamine.

Entry	Sample name	Chemical structure
1	MDI/BA-1	1,1′-[methylenebis(4,1-phenylene)]*bis*(3-benzylurea)
2	TDI/BA-2	a. 1,1′-(2-methyl-1,3-phenylene)*bis*(3-benzylurea) b. 1,1′-(2-methyl-1,5-phenylene)*bis*(3-benzylurea)
3	MDI/DBA-3	*N'N''*-[methylenebis(4,1-phenylene)]*bis*(*N,N*-dibutylurea)
4	TDI/DBA-4	a. *N'N''*-(2-methyl-1,3- phenylene)*bis*(*N,N*-dibutylurea) b. *N'N''*-(2-methyl-1,5-phenylene)*bis*(*N,N*-dibutylurea)

### Sample preparation procedure

In a 100 mL round bottom flask containing PA resin (20 g), the derivatizing agent (1 mL) and triethylamine (0.5 mL) were added dropwise at room temperature to avoid polymer precipitation. The sample was left to react under stirring for 12 h. Hexane (5 mL) and acetonitrile (10 mL) were added to precipitate the polymer avoiding the risk of clogging the instrumentation or altering the analytes. The mixture was stirred for 12 h and then left still for 1 h allowing the separation of the liquid phase (containing analytes) from the polymer matrix. This procedure was repeated for all real samples, maintaining under vigorous stirring. Then, an aliquot of 1 mL of the resulting solutions was filtered before HPLC-VWD analysis (Agilent™ PTFE filters 0.450 μm).

### HPLC-VWD analysis

The analysis was carried out by a HPLC apparatus (HPLC Agilent 1100 series, Agilent Technologies, Santa Clara, California, USA) using a Variable Wavelength Detector (VWD) detector set at 254 nm. The chromatographic separation was performed by using Luna column (C18, 150 × 4.6 mm) with 5 µm particle diameter (Phenomenex, Castel Maggiore, BO, Italy) and a temperature of 40 °C. The mobile phase was composed of solvent A, water and solvent B, acetonitrile according to an optimized gradient elution. The optimized gradient was not linear: 0 min, 50% B; 0–10 min 70% B, 10–20 min, 70% B, 20–40 min 80% B, 40–60 min 80% B; then the starting conditions were restored. The flow rate was set to 1 mL min^-1^, the injection volume was 10 µL.

The linearity was tested in the range 0.2–10 mg/l for MDI, and 1–40 mg/l for TDI, using external calibration standards. The regression coefficient R^2^ were 0.9995 and 0.9999 for MDI and TDI respectively. Sensitivity was determined on the base of the limit of detection (LOD) and limit of quantification (LOQ). The LOD was assessed as the concentration at which the signal (S) to noise (N) ratio is equal to three, instead the LOQ is associated to S/N = 10. MDI has 0.06 mg/l and 0.2 mg/l as LOD and LOQ respectively. TDI has 0.2 mg/l and 0.7 mg/l as LOD and LOQ respectively.

The precision of the overall method was evaluated by multiple analysis of real samples obtaining standard deviation in the range of 2.8–31.9%.

Accuracy was calculated on the base of the recovery values obtained by the analysis of the real sample as it is, and the same sample spiked with a known amount of MDI and TDI. Both samples were processed as independent ones, obtaining a recovery of 95.4% for MDI and 54.1% for TDI.

## Results and discussion

### DIs derivatization

**Figure 3:**
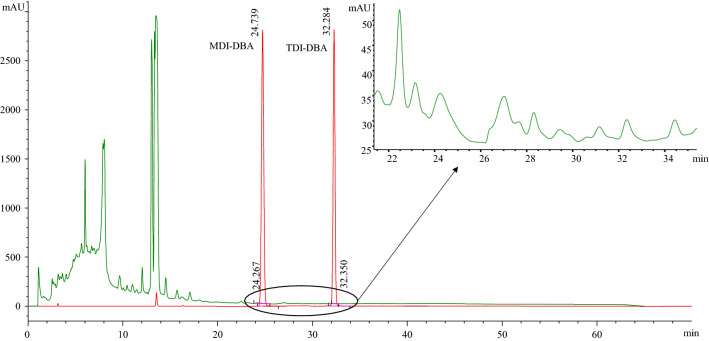
Overlay of a standard mixture (red line) and a real sample (green line) chromatograms.

DI compounds have reactive -NCO functions that must be stabilized before the HPLC-VWD analysis, giving rise to the necessity of derivatization for quantitative purposes. Secondary amines are commonly used as electrophilic derivatizing agents because the obtained urea derivatives are stable and quantifiable^28^. The advantages to perform the derivatization step were the increasing of the solubility in the HPLC mobile phase of derivatives, that is related to the chemical nature of the derivatization agent, and the improving of the chromatographic efficiency, avoiding coelution with components of the matrix. The latter is particularly important when a non-specific detector like UV is used. In this work two different amines were tested: an aromatic one such as BA, and an aliphatic amine like DBA. BA had the adding value of increasing the response of the correspondent derivative at the detection wavelength, due to the presence of an additional aromatic moiety able to respond at 254 nm. On the other side, the latter secondary amine had the advantage to improve the derivative solubility in the mobile phase. BA and DBA derivatives were synthetized and purified in our laboratories, then their solubility in the mobile phase used for the chromatographic separation was checked. Unfortunately, urea MDI/BA-1 was discarded due to its poor solubility in the eluent solution constituted by acetonitrile and water at a concentration of 1 mg/ml. The poor solubility could be explained due to presence of about 78% of NMP (NMP solvent for PAs matrixes). A subsequent step was testing the urea derivatives in the chromatographic condition in the presence of the matrix to evaluate detrimental overlap with matrix components. Urea TDI/BA**-**2 demonstrated to coelute with interfering species and cannot be used for quantitative purposes using UV detector system. Instead, both DBA derivatives of MDI and TDI revealed a good solubility in the mobile phase and retention times compatible with none of other signals coming from the matrix. For this reason, the PA samples were derivatized with DBA. In Fig. 3 is reported the overlay of chromatograms belonging to a standard mixture at 10 mg/l and a real sample. As can be seen, MDI-DBA and TDI-DBA have retention times of 24.7 and 32.4 min respectively, and their elution occur in correspondence of a chromatogram region without signals related to the matrix.

### Sensitivity of the method

Sensitivity is a crucial point for the quantification of residuals in complex matrices, specially when a derivatization step is necessary. In this case, sensitivity can be improved by different approaches: optimization of the derivatization yield, use of a chromophore able to increase the analyte detectability, achievement of a clear separation between derivatives and the matrix components, and the use of more specific and sensitive detectors. The goal can be reached by choosing the right strategy or more than one can be adopted at the same time, and the results strongly depends on the matrix composition. The LOQs of MDI reported in literature^24^ are 33.5 mg/l for HPLC–UV and 1.65 mg/l for HPLC–MS/MS when it is present in polyurethane foams and prepolymers. According to our results, we reached a lower sensitivity with values of 0.2 and 0.7 mg/l for MDI and TDI respectively. These findings can be rationalized taking in consideration that derivative species are clearly separated from the matrix components, originating signal to noise ratios similar to the standards matrix-free solutions. This fact could be addressed as the driving force to increase sensitivity and quantify very low level of MDI and TDI in solved-based varnish samples. In addition, the derivatization process occurs in high controlled anhydrous conditions, improving the derivatizing yields. Finally, variable wavelength detector (VWD) is the most sensitive among the UV detectors, even if we cannot have any confirmation of the analyte identity except for its retention time. Thus, all the listed precautions resulted in high level of sensitivity.

### DIs quantification in PAs samples

The developed analytical method was applied to PA samples manufactured by Elantas Europe S.r.l. company. Three batches produced in different seasons were analysed in order to evaluate the residual monomers MDI and TDI, in addition to the reproducibility of the industrial polymerization reaction. In fact, the high reactivity of DIs, especially in the presence of moisture in the reaction solvent, gave rise to a lack of standardization of the polymerization process. For this reason, it is a common practice to follow the polymerization reaction by monitoring the viscosity from time to time. If the viscosity, at the end of the production process, is under the stated specification, higher amount of monomers is used by subsequent addition. This is the case of the PA1 sample belonging to the batch produced in August 2020, that is labelled as anomalous. On the contrary, PA2 and PA3 samples were the results of a standard process. The results are reported in Table 3. Analyses revealed a residual amount of MDI in the range 0.3–1.7 mg/kg. Instead, higher concentrations of TDI monomer were found in all samples, registering concentrations between 9.1 and 28.4 mg/kg. The anomalous sample demonstrated levels of monomers comparable with the standard ones, indicating that the additional amount of monomers used during the process didn’t affect the quantity of residual monomers. Moreover, the residual monomers were largely lower of 2% w/w in all analysed samples, that avoid the registration activities according to the REACH Regulation.

**Table 3:** Concentration of MDI and TDI in real PAs samples expressed in mg/kg.

Sample		MDI		TDI	
		Mean conc.^a^ (mg/kg)	% RSD^b^	Mean conc.^a^ (mg/kg)	% RSD^b^
PA1	August 2020	1.7	31.9	9.1	2.8
PA2	January 2021	0.3	17.7	28.4	14.5
PA3	April 2021	1.0	7.0	9.5	3.0

^a^Mean concentration calculated as the average of three repeated analyses of the same sample.

^b^Relative standard deviation.

## Conclusions

In the present manuscript we developed a new and general analytical method able to determine residual monomeric aromatic isocyanates in many consumer products in which one of their primary raw material ingredients are diisocyanates. Although identifying and quantifying the content of aromatic isocyanates would be possible by several analytical methods, but each is highly specific and is labour intensive and expensive. Our method allows to analyse easily and quickly the common aromatic used-diisocyanates, such as toluene diisocyanate (TDI) and methylene diphenyl diisocyanate (MDI). For the first time, the optimized analytical method revealed good accuracy and precision over the concentration range 0.3–1.7 mg/kg of MDI, and 9.1–28.4 mg/kg of TDI resulting from the analysis of PAs real samples thanks to the derivatization method studied, through which it is possible to extract the analytes from the polymer matrix and analyze them.

The results obtained from this analysis indicate that the batch where an additional amount of monomers were added during the production process (labelled as anomalous) does not prove to have an abnormal concentration of diisocyanates compared to the other batches. In addition, there is no evident correlation between the storage time of the batch and the concentration of residual monomers in it. Therefore, it is logical to think that this new analytical method could be useful for further monitoring of the early-stage production in similar matrices as solvent-based polyamide-imides (PAIs) and polyamic acids (PAAs) which are easily synthesized by polycondensation of aromatic diamine with dianhydride, in order to avoid any hazard due to the high temperature used for their production in a “one-health” point of view. Furthermore, even more important is that the method finds application in viscous polymeric matrices, and therefore it suggests its use for the determination of residual isocyanates in medical devices^29^. In these consumer products complex analysis determinations of keratin-isocyanate adducts are required since isocyanates react instantly with skin components.

The method was studied for aromatic isocyanates because it is known that they are much more dangerous than the aliphatic correspondents^30^. Additional studies to improve this method, particularly regarding the development of an equally efficient and fast analytical method for aromatic and aliphatic isocyanates, are underway in our laboratories.

## Data Availability

The datasets generated during and/or analysed during the current study are available from the corresponding author on reasonable request.
